# Daily Encounters as a Measure of Accessibility: An Innovative Approach to Assessing the Influence of Gambling Landscape on Gambler's Behaviour

**DOI:** 10.1007/s10899-025-10392-0

**Published:** 2025-05-22

**Authors:** Filip Kovařík, David Fiedor, Jindřich Frajer, Miloslav Šerý, Miroslav Charvát, Eva Aigelová

**Affiliations:** 1 Department of Geography, Faculty of Science, Palacký University Olomouc, Olomouc, Czech Republic; 2 Department of Psychology, Faculty of Arts, Palacký University Olomouc, Olomouc, Czech Republic

**Keywords:** Availability, Accessibility, Encounter, Experience, Problem gambling

## Abstract

This study examines the relationship between daily encounters with gambling facilities and gambling participation in a Czech region that transformed from having one of the world's highest gambling facility densities to implementing strict regulations. Using a sample of 2,447 respondents from 277 municipalities, the research analyses how daily encounters with gambling facilities, rather than mere facility presence, influence gambling behaviour. The study incorporates individuals' accessibility based on daily encounters within their living space, moving beyond conventional metrics of availability. Results show that the extent of encounters during daily routines is a stronger predictor of gambling participation than facility presence alone, with the most pronounced effects observed in casinos and gaming halls. Municipalities with comprehensive gambling offerings showed the highest proportion of at-risk and problem gamblers. These findings emphasise the importance of considering population mobility in gambling accessibility analyses and suggest implications for local regulatory policies.

## Introduction

The relationship between the availability of services in residential areas and the frequency of their utilisation has been examined in various contexts. Focusing on activities that have a negative impact on human health, studies have investigated the physical availability of alcohol in relation to increased consumption (Chaloupka & Wechsler, 1996; Jones-Webb et al., 1997), with marketing and promotion of alcoholic products also playing a significant role (Sureda et al., 2017). Research has extensively documented that higher tobacco outlet density is associated with increased smoking prevalence (Shortt et al., 2016), higher tobacco use and smoking initiation, and reduced cessation success (Pearce et al., 2015; Valiente et al., 2021). High smoking prevalence is also associated with the normalisation of smoking and smoker visibility in urban areas (Valiente et al., 2020). Similarly, research has shown that the proximity of fast-food restaurants is associated with increased tendencies towards obesity (Rundle et al., 2009). The results of these studies are essential for decision-makers and authorities who can regulate some of the services at different scales (local, regional/state, federal) with political and economic instruments to mitigate their adverse impacts on human health. This also applies to the issue of gambling accessibility, which is the focus of this study. The accessibility of gambling venues is a crucial factor in investigating risks associated with problem gambling (Pearce et al., 2008; Vasiliadis et al., 2013). Research on this topic has demonstrated that higher spatial accessibility of gambling opportunities correlates with increased participation among residents (Pearce et al., 2008), as well as a higher incidence of gambling-related problems that may arise from increased participation (Parrado-González et al., 2023). Given the wide range of harms associated with problem gambling (financial, psychological, emotional, and social, see Sulkunen et al., 2020), regulation of advertising, taxation, but also accessibility, availability, and geolocation of gambling products can play an important role (The Lancet, 2023). Gambling activities are legal in 85% of the world's countries. Still, the degree and manner of regulation varies considerably regionally (Ukhova et al., 2024), not only between countries in the same region but also between municipalities (Fiedor et al., 2019a), urban districts, or is restricted to unique locations (Yorifuji, 2005). Thus, the legislative framework can create a time-varying'gambling landscape'within which locations with different concentrations of gambling facilities tied to specific types of gambling activities exist side by side. Thus, people visit various places of the imagined'gambling landscape'with different availability of gambling activities as part of their daily movements, which can affect their participation in gambling. This aspect is the focus of our research.

### Accessibility and Availability of Gambling Facilities: A Methodical Gap

In the context of population exposure to various types of gambling facilities, two distinct terms are employed:"accessibility"and"availability", which are sometimes misconstrued as synonymous (Productivity Commission, 2010). Ofori Dei et al. (2021), however, caution that whilst these terms are interrelated, they represent distinct concepts. Whereas"availability"typically focuses on the presence of facilities and may be understood in binary terms (as presence or absence of gambling facility in the selected spatial unit),"accessibility"encompasses more complex aspects, such as the ease with which one can physically reach a facility. Moore et al. (2011) corroborated that gambling accessibility is a multidimensional concept encompassing both social and physical aspects. In this context Hing and Haw (2009) identified three primary dimensions of accessibility in their study: social (approbation of gambling from personal, familial and peer sphere), physical (diversity of options, venue proximity, minimal waiting time), and cognitive (proficiency in and comprehension of gambling product mechanics). Despite the significant role of social accessibility, the predominant focus of research is on physical accessibility, which can be effectively studied with the use of spatial analysis in Geographical Information Systems (Saunders et al., 2023; Selin et al., 2024). Within gambling studies, both availability (Wardle et al., 2014) and accessibility (Young et al., 2012a) have been examined. Welte et al. (2004) report that individuals residing within 10 miles of a casino exhibit twice the rate of pathological or problem gambling compared to those living further away. Subsequent research confirmed increased participation and risky gambling behaviour among residents living up to 30 miles from a casino and also established a relationship between the proximity of residence to horse and dog racing tracks and frequent gambling in those activities (Welte et al., 2015). Moreover, Wardle et al. (2014) demonstrated a strong correlation between socioeconomic deprivation and the density of electronic gambling machines (EGMs), with zones characterised by a high density of gambling machines (HDMZ) housing more economically inactive individuals who are, on average, younger and more vulnerable to the negative impacts of gambling. In a similar fashion, Selin et al. (2024) revealed the concentration of EGMs gambling in neighbourhoods with a lower socioeconomic status. Recent research has also highlighted concerns regarding the spatial distribution of betting shops in urban areas, particularly their proximity to sensitive locations such as schools. For instance, Arias Rodríguez and Escobar (2025) identified areas with high density of betting shops in Madrid, Spain, where a significant number of schools were exposed to these facilities. The accessibility of gambling is a significant factor. Young et al. (2012a) emphasise that it should be considered one of the critical parameters for assessing the social impacts of gambling on communities within regulatory policies.

St.-Pierre et al. (2014), in their review study, posit that whilst the relationship between the geographical availability of gambling venues and increased gambling participation is logical, the connection with a higher incidence of problem gambling may not be as straightforward. The utilisation of statistical data to establish theoretical availability/accessibility of gambling facilities using various criteria (total number of gambling venues, density of facilities, or buffer zones) is confined to a specific spatial unit (administrative or statistical). This approach faces what has been termed the'uncertain geographic context problem'(Kwan, 2012), where conventional measures based on fixed and static areal units cannot adequately capture the true geographic context of individuals who move through different spaces over time (Chun et al., 2019). It may not fully capture the complexity and differences in whether residents living in this spatial unit are actually exposed to gambling facilities during their daily movements, for instance, to work, schools, sports activities, or shopping. Advances in time-geographic research and GIS methods (Kwan, 2004) offer promising approaches for better understanding these individual mobility patterns and their intersection with gambling opportunities. Moreover, these movements and activities may be linked to spatial units other than those in which people reside. The importance of considering both spatial and temporal dimensions of human mobility in understanding accessibility has been emphasized in geographical research (Kwan & Schwanen, 2016), suggesting that traditional static measures of gambling accessibility may be insufficient. This is particularly evident in Young et al.'s (2012b) findings of increased participation and a higher prevalence of problem gamblers in EGMs venues located in proximity to supermarkets. We posit that the question of the extent to which individuals routinely encounter these facilities and the impact this has on their participation in gambling activities remains an insufficiently explored aspect of research into the role of geographical accessibility within gambling studies. Consequently, gambling exposure may play an equal, if not more significant, role in people's decisions to engage in gambling than mere availability. Suppose a person lives in a city where gambling facilities exist but is not exposed to them. In that case, their likelihood of engaging in gambling may be lower than that of a person who regularly encounters these facilities, regardless of whether they live in that city or not.

Based on the findings mentioned above and identified research gaps, this study aims to determine the significance of daily exposure to different gambling segments on gambling participation and the prevalence of problem gambling. This approach overcomes the limitations of traditional measurements of actual availability and considers individuals'subjective experiences with gambling in their daily lives and lived space. To achieve this objective, it will be necessary to compare the proposed predictor with the traditional physical availability of gambling facilities at the level of administrative units. For these research purposes, we chose the territory of the Olomouc region in the Czech Republic.

### Geographical Background

The gambling industry in the Czech Republic has undergone a profound transformation since 1989. During the communist regime, gambling operations were strictly limited to state-controlled casinos, predominantly situated in metropolitan areas and spa resorts. Following the dissolution of the communist regime in 1989, the landscape altered dramatically, with the Czech Republic progressively evolving into one of the nations with the highest density of gambling devices globally (Frajer et al., 2024). A pivotal regulatory milestone was established through Act No. 202/1990 Coll. on Lotteries and Similar Games, which remained substantially unaltered until 2012. A significant legislative advancement materialised with Amendment No. 300/2011 Coll., which implemented a more transparent system of financial levies and delegated regulatory authority over gambling facilities to municipal governments. Civil society organisations played an instrumental role in advancing these regulatory measures by catalysing public discourse on gambling-related issues (Fiedor et al., 2019a). As a consequence of these regulatory interventions, the number of land-based gambling facilities decreased substantially – from 8,367 in 2011 to merely 707 in 2023. Correspondingly, the quantity of Electronic Gambling Machines (EGMs) diminished from 102,000 to 24,000 during the same period. Within this context of dramatic changes in the Czech gambling landscape, it is particularly important to understand how accessibility and exposure to gambling facilities influence participation rates (Fiedor et al., 2024).

## Materials And Methods

### Database of All Gambling Facilities

The Czech Republic is among the post-socialist countries with very good accessibility to a wide range of gambling activities. However, official data on the number and location of land-based gambling facilities (availability) are collected and published by the Ministry of Finance only for so-called hard forms of gambling, specifically electronic gambling machines and casino table games (Chomynová et al., 2023). Data on other forms of land-based gambling, including sports betting shops and lottery outlets (including scratch cards), are published only as part of individual operators'promotional activities. The Ministry thus maintains only a list of legal land-based gambling operators, not individual facilities. For sports betting, this list contained four companies^1^ at the end of 2023, whilst for lotteries, there were six companies^2^ in total. To work with a comprehensive list of all gambling-related locations, official statistics had to be supplemented with data from individual companies'websites. Information on the location of individual places of sale was thus obtained, based on which a point layer for GIS analysis was manually created.

The Olomouc region was selected as a model region for a comprehensive analysis of gambling exposure to its heterogeneous composition, encompassing urban centres, rural zones, and outlying territories (Fiedor et al., 2019b). The region is also appropriate for examining gambling, as it houses a substantial casino complex that conducts major card competitions, alongside other gambling facilities. Additional gambling opportunities, such as sports betting shops and lottery outlets, are also present (Table 1). Conversely, gaming activities are notably sparse in the remote sections of the region (Frajer et al., 2024).

**Table 1:** Comparison of Absolute and Relative (per 10,000 Adult Inhabitants) Numbers of Facilities of Individual Gambling Segments in the Model Area of the Olomouc Region and the Czech Republic

Gambling Segment	Olomouc Region		Czech Republic	
	ABS	PER 10,000	ABS	PER 10,000
Lottery	715	13.9	10,721	12.2
Sports Betting	134	2.6	1,611	1.8
Gambling Halls^a^ and Casinos	45	0.9	714	0.8
Thereof: Slot Machines	1,525	29.7	24,282	27.6
Thereof: Live Casino Aames	228	4.4	3,310	3.8

CZSO (2022), MFCR (2024), legal land-based gambling operators’ websites; own processing

^a^Also referred to as gambling rooms or gaming halls

When examining the total numbers of lottery outlets, sports betting shops, and gambling halls and casinos, it is evident that lotteries are the most accessible, whilst gambling halls and casinos are the least widespread. This corresponds to the varying levels of risk for developing problem gambling (Kovařík & Fiedor, 2025), where lotteries are considered the least risky (Binde et al., 2017), sports betting represents a medium level of risk (LaPlante et al., 2014), and casino games, particularly EGMs, are the most risky (Williams et al., 2021). This raises the question of whether Christaller's central place theory (Christaller, 1933) might be applicable here, describing a hierarchical structure of settlements where higher-order centres provide a more comprehensive range of services and serve larger territories than lower-order centres.

With one exception (the municipality of Černotín^3^), this holds true, meaning that in a municipality where a gambling hall or casino is located, there is also a sports betting shop and a lottery outlet. Similarly, in a municipality with a sports betting shop, there is also a lottery outlet. Considering this distribution, four categories of municipalities can be established:

1. Municipality with all types of gambling segments (hereafter referred to as Casino Game Municipality = CGM);
2. Municipality with a sports betting shop and a lottery outlet (hereafter referred to as Betting Municipality = BtM);
3. Municipality with only a lottery outlet (hereafter referred to as Lottery Municipality = LtM);
4. Municipality without any gambling facility (hereafter referred to as Non-Gambling Municipality = NGM).

### Questionnaire

To investigate the accessibility of gambling, our research methodology incorporated a survey instrument aimed at mature individuals^4^ in the Olomouc region. Our survey instrument was disseminated via two methods: physical copies (PAPI) distributed on-site (June 2023) and a digital version accessible from July through December 2023. The in-person data collection was facilitated by university scholars who underwent preparatory training before visiting diverse localities within the designated area. Following this, we catalogued the mayors of all municipalities in the region and employed a dual-phase outreach strategy, soliciting their assistance in circulating the questionnaire through official web portals and social media platforms. Of the 402 total mayors, 123 (31%) cooperated. Given the demanding nature of their roles (Fiedor et al., 2019a), this level of engagement is considered substantial. The survey's reach was further amplified by governmental bodies and local media sharing the digital link. This multi-faceted distribution strategy ensured broad coverage across the region, resulting in representation from 277 municipalities (69% of the total).

The survey instrument incorporated demographic inquiries, encompassing sex (options: male, female, or alternative), participant's age, the highest level of education obtained, and current residential locality. Content-wise, it examined, using a dichotomous variable (yes x no), whether respondents encountered a) a gambling hall or casino, b) a sports betting shop, and c) a lottery outlet on their regular movements. Furthermore, personal experience with various gambling activities^5^ was investigated (on an ordinal scale: never/sometime in life/in the last year/in the last month). The survey culminated with a concise evaluation tool designed to assess potentially harmful gambling behaviours based on the experience in the last 12 months. We utilised the PGSI Short form (Volberg & Williams, 2012), a validated instrument comprising three key inquiries. Participants responded to each statement using a quartet of frequency options, ranging from"never"(scored as 0),"sometimes"(1),"most of the time"(2), to"almost always"(3). By aggregating these individual scores, we derived a cumulative metric. This aggregate score then facilitated the classification of respondents into three distinct risk categories (Dowling et al., 2018): those exhibiting no problematic behaviour (0 points), individuals at-risk (1–2 points), and persons meeting the criteria for problem gambling (3 points or more).

Ethical clearance for this study was granted by the Ethics Panel of the Faculty of Arts at Palacký University Olomouc (standard FF-B-20–02, registration number 02/2023). The data acquisition process ensured complete anonymity, and the subjects'involvement was strictly voluntary. Informed consent was obtained from all participants prior to their engagement in the study. For field-based data collection, verbal consent was deemed sufficient, whilst, in the online environment, consent was indicated by the participant's action of selecting the'complete questionnaire'option. Our data collection effort yielded 2,724 survey responses. The subsequent data refinement process led to the exclusion of 277 entries, representing 5.7% of paper submissions and 15.2% of digital responses. These exclusions were necessitated by various factors: incomplete or erroneous submissions (127 cases), participants below the gambling legal age (55 instances), respondents outside the Olomouc Region (44 occurrences), and failure to specify residential locality (51 cases). Post-refinement, our analytical dataset comprised 2,447 valid responses, with 1,366 (55.8%) originating from paper-based surveys and 1,081 (44.2%) from online submissions. The demographic composition of our final sample included 1,025 male participants (41.9%), 1,407 female participants (57.5%), and 15 individuals (0.6%) who either selected'other'or abstained from gender specification. Participants'ages ranged from 18 to 93 years, with a mean of 43.1 years and a median of 41. Our sample constitutes approximately 0.49% of the adult populace within the investigated region, as detailed in Table 2.

**Table 2:** The Relationship Between the Hierarchy of Municipalities in the Olomouc Region Concerning the Availability of Gambling Segments, Supplemented by the Structure of Respondents

	Municipalities	Adult Population (18 +)	Municipalities Involved in the Research	Number of Respondents	Proportion of Respondents Amongst the Adult Population
Total	402	503,685	277	2,447	0.49
Thereof: NGM	215	70,474	125	448	0.64
Thereof: LtM	187	433,211	152	1,999	0.46
Thereof: BtM	50	314,010	43	1,431	0.46
Thereof: CGM	13	241,864	13	1,110	0.46

CZSO (2022), MFCR (2024), legal land-based gambling operators’ websites; own processing

### Statistical Analysis

In addition to the basic methods of descriptive statistics, hypothesis testing was used. Specifically, this involved the use of the chi-square test and the non-parametric Kruskal–Wallis test (K-W test) for multiple group comparisons. All tests were evaluated at the α = 0.05 level of significance. With respect to the type of variables, ordinal logistic regression was used to explain the variability in the problem gambling rate (ordinal variable). Problem gambling was assessed using the PGSI categories (described above), with the PGSI Short form being completed only by respondents with gambling experience in the past year. Thus, the analysis focuses on explaining the level of problem gambling among those with relatively recent experience. We used R (version 4.4.3; R Core Team, 2025) with the MASS package (Version 7.3–65; Ripley et al., 2025) for ordinal logistic regression and the rcompanion package (Version 2.5.0; Mangiafico, 2025) for calculating model fit statistics.

## Results

### Spatial Differentiation of Gambling Facilities and Daily Encounter

**Fig. 1:**
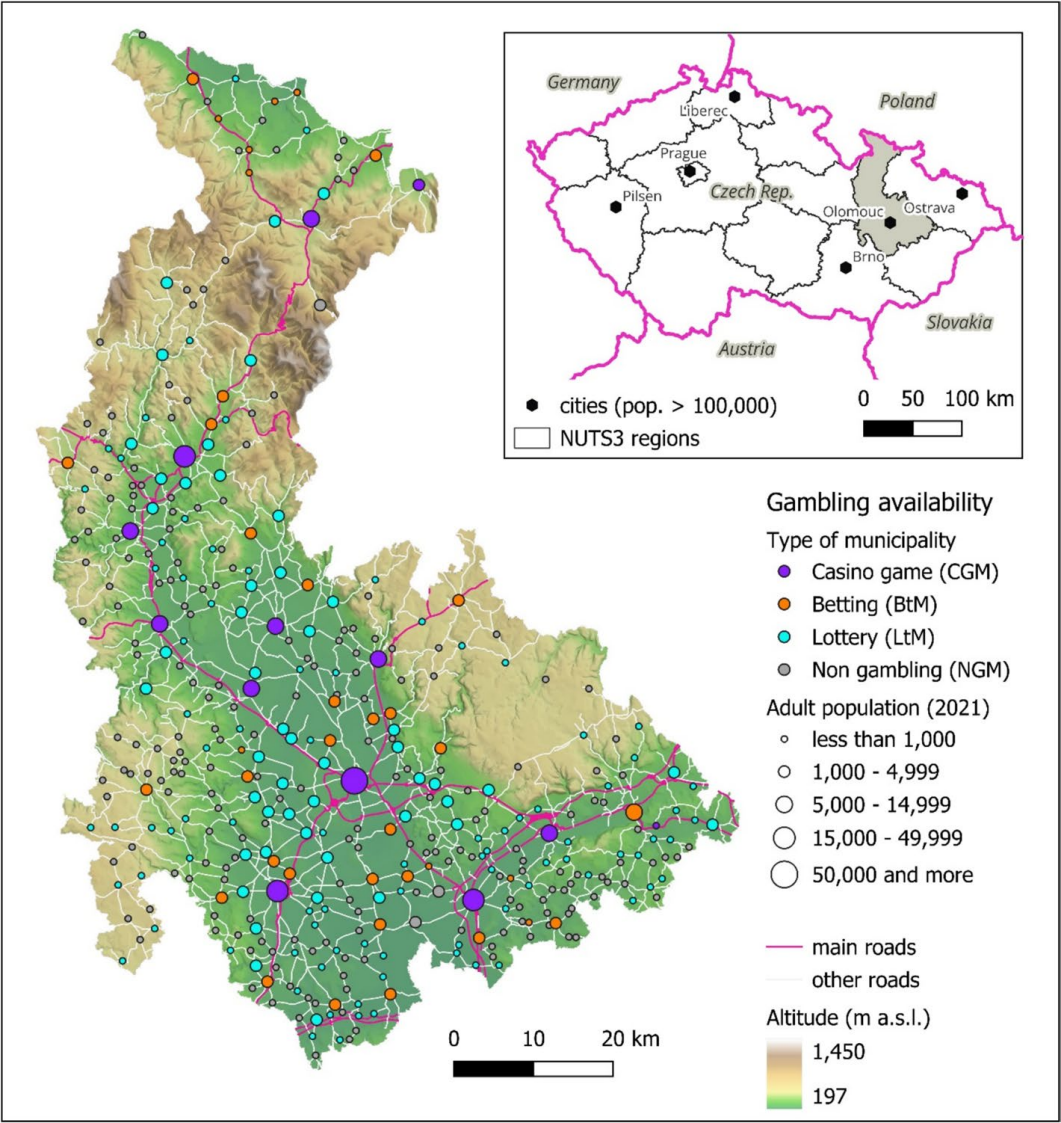
Types of Municipalities According to the Availability of Gambling Segments ‘ Facilities in the Olomouc Region. Source: CZSO (2022), MFCR (2024), legal land-based gambling operators’ websites, State Administration of Land Surveying and Cadastre (2024); own processing

As shown in Fig. 1, the occurrence of municipalities with gambling segments in the area of interest is unevenly distributed, which is partly due to the settlement system. The topographical background of the map was deliberately included to illustrate how terrain characteristics influence both the settlement pattern and transport accessibility. The area with relatively abundant gambling in the central and southern parts, led by the regional capital (city of Olomouc), is adjacent to peripheral areas in the north (mountainous region), west and east (influenced by military districts), where there is both a lower concentration of municipalities and a more miniature representation of gambling. This spatial pattern clearly demonstrates how challenging topography contributes to the peripherality of certain areas, affecting both the distribution of settlements and the availability of gambling services. The representation of individual gambling segments in municipalities is primarily linked to the population size of the municipality and its transport accessibility. Casino Game Municipalities (CGM) are predominantly tied to more populous municipalities, with 84.6% having more than 5,000 adult residents, and at the same time, they are located near important road communications. Betting Municipalities (BtM) are concentrated in proximity to CGMs, with a predominance of settlements in the category of 1,000 to 5,000 adult residents (72.9%). Their connection to significant road routes is particularly evident in peripheral areas. In the case of the most widespread type, Lottery Municipalities (LtM), 64.2% are municipalities with up to 1,000 adult residents. Of all municipalities without gambling activities, only 1.4% fall into the category of over 1,000 adult residents.

The presence of a gambling facility in a municipality does not necessarily mean that an individual comes into contact with it. For this reason, the questionnaire inquired whether respondents encounter these facilities during their daily movements (commuting to work and shopping). Evidently, a facility's presence in a municipality significantly influences daily exposure. For all three segments of interest (gambling halls and casinos, sports betting, and lottery), those residing in municipalities where these facilities are present encounter them to a greater extent (Table 3). The most substantial difference is observed for gambling halls and casinos (22.7 percentage points), while the smallest difference is for lottery outlets (9.2 percentage points). This clearly correlates with their spatial distribution; the more common the gambling segment, the smaller the difference in the proportion of individuals who regularly encounter it. For lotteries, a situation arises where more than half of the respondents regularly encounter these facilities regardless of whether they are present in their municipality of residence. The situation is reversed for gambling halls and casinos, of which there are only 45 in the area of interest, i.e., 0.9 per 10,000 adult residents (Table 1). In municipalities where these facilities are present, only 48.1% of respondents regularly encounter them, and in municipalities where they are absent, this figure drops to 25.4%. For all three gambling segments, the relationship between the presence of a facility in the municipality and encounters is statistically significant at the α = 0.05 level of significance.

**Table 3:** Encountering Facilities of Individual Gambling Segments by Respondents According to the Actual Availability of a Given Type of Facility in Their Municipality of Residence

Encounter/Available	Gambling Halls and Casinos		Sports Betting		Lottery	
	Yes	No	Yes	No	Yes	No
Yes	48.1	25.4	57.0	41.6	66.1	56.9
No	51.9	74.6	43.0	58.4	33.9	43.1
Total	100.0	100.0	100.0	100.0	100.0	100.0
	(N = 1,110)	(N = 1,337)	(N = 1,431)	(N = 1,016)	(N = 1,999)	(N = 448)
Chi-square Test	p-value = 0.000		p-value = 0.000		p-value = 0.000	

MFCR (2024), legal land-based gambling operators’ websites; own processing

### The Impact of Availability and Encountering of Gambling Facilities on Participation

It is evident that daily encounters are positively influenced by the presence of gambling facilities in the respondent's municipality of residence. However, the main objective of this paper is to identify how varying degrees of accessibility to gambling facilities affect participation in gambling activities and, subsequently, potential problematic gambling behaviour. This analysis will enable us to understand better the complex interaction patterns between the spatial context of gambling and population participation. Overall, it can be stated that the population has the most experience with the most accessible segment of gambling, i.e., lotteries. Only 14.2% of respondents have no experience with lotteries. In contrast, only 16.9% of respondents have some experience with sports betting in land-based outlets, while 34.9% have experience with casino-type games.

**Fig. 2:**
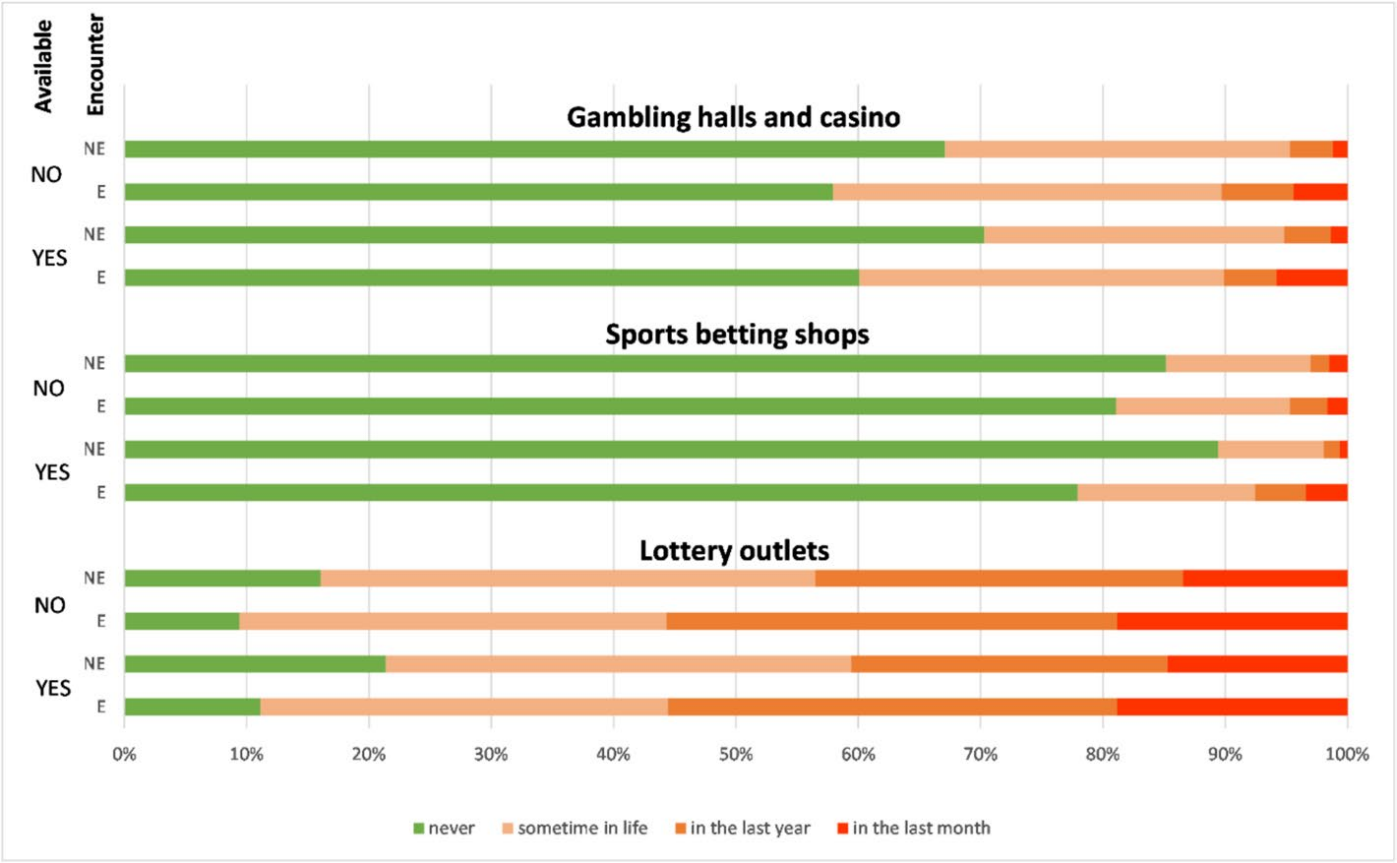
Encounters with Individual Segments of Gambling Based on the Availability of the Given Gambling Facility. Source: MFCR (2024), legal land-based gambling operators’ websites; own processing

Examining Fig. 2, it is clear that the decisive factor for participation in all segments of gambling is encountering facilities during one's regular movements. For casino-type games, those who encounter them have more experience (regardless of whether gambling halls and casinos are present in their municipality of residence). In this regard, it can be stated that using the K-W test and subsequent multiple comparisons, statistically significant differences were demonstrated at the α = 0.05 significance level between all pairs of categories,^6^ except for two:"NO/NE"with"Y/NE"and"Y/E"with"NO/E". For all others, the relationship was statistically significant: 1)"NO/E"with"NO/NE"(p-value = 0.022), 2)"NO/E"with"Y/NE"(p-value = 0.004), 3)"Y/E"with"NO/NE"(p-value = 0.034) and 4)"Y/E"with"NO/NE"(p-value = 0.006). When looking at experiences in the last month, the main difference is again the factor of encountering, not the presence of a facility in the municipality. Nevertheless, it appears (Fig. 2) that among those who encounter gambling halls and casinos and live in a municipality where these facilities are present, 5.8% of respondents have experience with this segment of gambling. In contrast, this proportion is slightly lower for those who do not live in a municipality with these facilities but encounter them (4.4%). For other gambling segments, the situation is similar, i.e., encountering gambling facilities is associated with a higher rate of participation and a higher proportion of respondents who have experience in the last month. Multiple comparisons showed a statistically significant difference for sports betting shops only between the categories"Y/E"and"Y/NE"(p-value = 0.001). Differences between all other pairs were not statistically significant at the α = 0.05 significance level. For lottery outlets, significant differences were demonstrated between three pairs: 1)"Y/E"with"Y/NE"(p-value = 0.000), 2)"Y/E"with"NO/NE"(p-value = 0.015), and 3)"NO/E"with"Y/NE"(p-value = 0.000). These results indicate that regular encounters with gambling facilities during routine daily movements are a stronger predictor of participation than the mere presence of a facility in the municipality of residence.

### Problem Gambling in Relation to Municipality Type and Encountering Gambling Facilities

A total of 1,366 respondents (55.8%) had some gambling experience in the past year. Given that 161 respondents did not complete the PGSI Short form,^7^ in the following analyses the authors work with the 1,205 respondents who had gambling experience in the past year and completed the PGSI Short form. The availability of various gambling segments can be a particular predictor of problem gambling (Table 4). In municipalities with a comprehensive range of gambling segments (CGM), including casinos and gaming halls, sports betting shops, and lottery outlets, we observe the highest cumulative proportion of at-risk and problem gamblers (26.7%). This proportion gradually decreases with the declining presence of gambling facilities in the municipality, with the lowest value (15.2%) observed in municipalities without gambling facilities (NGM). According to the Chi-square test, the association between the type of municipality in which the respondent lives and the problematic nature of their gambling is statistically significant at the α = 0.05 significance level (p-value = 0.023).

**Table 4:** Prevalence of Problem Gambling Categories Depending on the Type of Municipality According to Gambling Facilities Availability

PGSI Short form	CGM	BtM	LtM	NGM	Total
Non Problem Gambling	73.3	75.4	80.9	84.8	77.4
At-Risk Gambling	18.0	16.2	13.6	10.3	15.4
Problem Gambling	8.7	8.4	5.5	4.9	7.2
Total	100.0	100.0	100.0	100.0	100.0
	(N = 543)	(N = 167)	(N = 272)	(N = 223)	(N = 1,205)

MFCR (2024), legal land-based gambling operators’ websites; own processing

Although the availability of a wide range of gambling segments is, to some extent, a factor, it has already been pointed out in terms of participation rates that physical accessibility measured through daily encounters is a better predictor. The same holds true for the problematic nature of gambling (Table 5). Among respondents who encounter gambling halls and casinos during their regular movements, 29.9% are classified as being at some risk of developing problem gambling, and 12.0% can already be considered problem gamblers. The lowest proportion of problem gamblers can be found among respondents who do not encounter any gambling facilities (4.4%) and those who do not encounter gambling halls and casinos (4.4%). The more accessible the gambling segment, the greater the difference in the proportion of problem gamblers who encounter facilities of that segment and those who do not. This is because each segment is always evaluated separately, meaning that for those who encounter lottery outlets, it is unknown whether they also encounter gambling halls and casinos. Different segments of gambling have varying impacts on the development of problem gambling. In all cases (except for encountering lottery outlets), the Chi-square test demonstrated a statistically significant relationship between encountering the facility of a given gambling segment and problem gambling at the α = 0.05 significance level.

**Table 5:** Distribution of Problem Gambling Risk Based on Encounters with Various Gambling Segments

PGSI Short form	Gambling Halls and Casinos		Sports Betting		Lottery		Any of These	
	E	NE	E	NE	E	NE	E	NE
Non Problem Gambling	70.1	81.8	74.0	81.5	78.1	75.8	75.8	86.3
At-Risk Gambling	17.9	13.8	17.3	13.1	15.2	15.7	16.5	9.3
Problem Gambling	12.0	4.4	8.7	5.5	6.7	8.5	7.7	4.4
Total	100.0	100.0	100.0	100.0	100.0	100.0	100.0	100.0
	(N = 452)	(N = 753)	(N = 655)	(N = 550)	(N = 854)	(N = 351)	(N = 1,022)	(N = 183)
Chi-square Test	p-value = 0.000		p-value = 0.007		p-value = 0.493		p-value = 0.007	

MFCR (2024), legal land-based gambling operators’ websites; own processing

Explaining the level of problem gambling itself is more complex, as many factors need to be taken into consideration. The ordinal logistic regression (Table 6), which incorporates a limited number of factors, namely segment encounters, type of municipality in terms of presence of each type of facility, gender and age, explains 15.1% of the variability in the dependent variable (Nagelkerke's R^2^ = 0.151), and the model is highly statistically significant (χ^2^(8) = 168.24, *p* < 0.001). Encounters with casinos (OR = 1.73) and betting shops (OR = 1.40), gender (OR = 4.15), and age (OR = 0.99) are significant predictors of higher gambling risk. Individuals encountering casinos frequently have 1.73 times higher odds of being in a higher PGSI category, those encountering betting shops have 1.40 times higher odds, men have more than 4 times higher odds of being in a higher PGSI category compared to women, and older age is associated with slightly lower odds of being in a higher PGSI category. The type of municipality shows significant effects, particularly in betting-dense areas (OR = 2.11) and casino-dense areas (OR = 1.69), while lottery encounters were found to be non-significant.

**Table 6:** Ordinal Logistic Regression for the Dependent Variable Problem Gambling (PGSI Category)

Predictor	Estimate	SE	Z	p	Odds Ratio
Encounters Lottery:					
1–0	−0.2651	0.17219	−1.540	0.124	0.767
Encounters Betting:					
1–0	0.3366	0.16524	2.037	0.042	1.400
Encounters Casino:					
1–0	0.5491	0.15303	3.588	<.001	1.732
Type of Municipality:					
LtM – NGM	0.3492	0.25469	1.371	0.170	1.418
BtM – NGM	0.7467	0.27327	2.733	0.006	2.110
CGM – NGM	0.5226	0.22679	2.304	0.021	1.686
Gender:					
Man – Woman	1.4229	0.15709	9.057	<.001	4.149
Age	−0.0129	0.00491	−2.619	0.009	0.987

MFCR (2024), legal land-based gambling operators’ websites; own processing

## Discussion

In relation to gambling availability, the study confirmed the association of casino games with urban environments, specifically larger population centres. Lambert et al. (2010) noted that casinos tend to perform better economically when located in metropolitan area centres compared to those situated in less populated regions. Similarly, Kristiansen and Lund (2022) identified the presence of larger clusters of electronic gaming machines (EGMs) in connection with major cities in their research conducted in Denmark. In this context, the principle of central place theory can be considered, where casino games constitute a high-order service within gambling activities, linked to larger population centres (in our case, CGM) to cover the largest possible number of inhabitants or customers (Zhao et al., 2023). Medium-order services, such as sports betting, are associated with smaller population centres (BtM), and in the smallest municipalities, only lotteries (LtM) are present, which are the most accessible form of gambling in the Czech Republic due to their distribution through small shops, newsstands, or post offices (Fiedor et al., 2019b). In line with central place theory, the condition of availability of all low-order gambling"services"was confirmed in the studied area, except for one municipality (out of a total of 402) within higher-order centres. Thus, sports betting and lotteries were also available within CGM, and lotteries were available within BtM.

Larger municipalities with high or medium-order gambling facilities (CGM, BtM) are also centres of functional regions determined by regular daily movements, whether for work or school (Halás et al., 2021). This also explains why a quarter of respondents in our research reported encountering gambling halls or casinos on their usual routes, even if they were not available in their place of residence. Similarly, 41% of respondents encountered sports betting outlets, and nearly 57% encountered lotteries despite their unavailability in their place of residence. Although the availability of gambling facilities is a predictor associated with higher participation rates, as confirmed by previous studies (Pearce et al., 2008; Vasiliadis et al., 2013; Welte et al., 2004, 2016), it did not prove to be as strong a factor as gambling encounters. This factor highlights the experiential dimension of gambling exposure, allowing for the capture of the actual level of exposure individuals have to gambling facilities in the context of their daily spatial movements, providing a more detailed picture of the real interaction of residents with the gambling landscape. Through this methodological approach, we have also mitigated the Modifiable Areal Unit Problem (MAUP), a challenge in spatial analyses arising from artificially delineated boundaries (Wong, 2004). By focusing the research on encounters with gambling facilities, potential biases arising from subjective perceptions of these facilities and cognitive biases associated with their perception may have occurred. Respondents utilised their mental maps (Tuan, 1975) when answering questions about encountering gambling facilities. Our data collection relied on respondents'declarations based on retrospective ex-post recall of their spatiotemporal activities (Brisudová et al., 2024). This methodological limitation could be mitigated by, for instance, having respondents create space–time diaries, providing more accurate and real-time data on facility encounters. Consequently, while our approach yielded valuable insights, our research is probably, to some extent, subject to cognitive or mental bias. The questionnaire was also oriented towards the spatial mobility of residents during the daily cycle rather than the direct residential localisation of respondents. This approach avoided the need to collect sensitive personal data about the exact address of the respondent's residence, which can reduce response rates and increase reporting errors (Tourangeau & Yan, 2007). We believe that our chosen indicator of gambling accessibility can encompass the issue of daily commuting and, thus, exposure to gambling in a city other than the place of residence.

As LaPlante and Shaffer (2007) argue, exposure does not necessarily lead directly to gambling addiction and problem gambling. The connection between encountering individual facilities and problem gambling was confirmed for gambling halls and casinos, sports betting shops, and encounters with any gambling facility, but not for encounters with lottery outlets. The highest proportion of problem gamblers is found among those who encounter gambling halls and casinos on their usual movements, while the lowest proportion is among those who do not encounter any gambling facilities. This can be explained by the fact that different forms of gambling represent varying levels of risk for developing problem gambling (Mravčík et al., 2020). The potential riskiness of individual gambling segments corresponds to their availability, with lotteries, for example, being the most accessible as a less risky segment (Ariyabuddhiphongs, 2011; Garrett, 2001). Although a statistically significant relationship between encountering lottery outlets and problem gambling was not established, the proportions of problem gamblers in both groups (among those encountering lottery outlets and among those not encountering them) are notably higher than among those who do not encounter any gambling facility. From a prevention perspective and within the public health approach, experts recommend focusing on the entire gambling population (Livingstone & Rintoul, 2020), as gambling is always a risky activity. This approach aligns with the options available to local governments in regulating gambling. In the Czech Republic, municipalities have the legislative authority to regulate the operation of gambling within their administrative territory through generally binding ordinances. These legal acts allow local governments to implement regulatory measures, which may include a blanket prohibition of gambling or its territorial limitation to designated locations within the municipality. This form of regulation can induce the relocation of gambling facilities outside the municipality's built-up area, potentially leading to a reduction in the frequency of gambling encounters among the population. These regulatory interventions aimed at relocating gambling facilities to less frequented parts of the municipality can lead to a decrease in the prevalence of participation and problem gambling in the population. However, as research among local government representatives indicates, the tolerance for hard forms of gambling increases with the size of the municipality, particularly in connection with the economic benefits for the municipality, especially from taxes (Fiedor et al., 2019a). On the other hand, it is also necessary to consider factors such as the possibility of commuting to gamble in other locations (Greenwood & Dwyer, 2017) or participation in online gambling (Gainsbury et al., 2015).

While our study provides valuable insights into the relationship between encounters with gambling venues and gambling behaviour, we acknowledge certain methodological limitations. Although GPS-based technologies could offer more precise spatial–temporal data about human movement patterns and activities in urban settings (Van der Spek et al., 2009), including potential encounters with gambling venues, we opted for a questionnaire-based approach. This decision was primarily driven by two considerations. Firstly, GPS tracking methods, whilst providing more granular data, could significantly limit our sample size due to their demanding nature for participants and potential selection bias. Our questionnaire-based methodology enabled us to collect data from a more robust and diverse sample. Secondly, GPS tracking raises substantial ethical concerns regarding privacy and surveillance, particularly in the context of gambling research where data sensitivity is heightened. These ethical considerations, combined with our aim to capture a broader representative sample, justified our methodological choice. Another limitation of our study relates to the self-reported nature of gambling venue encounters. We acknowledge that individuals with a pre-existing interest in gambling may be more attentive to and better recall gambling-related environmental cues, potentially creating a self-fulfilling relationship between reported encounters and gambling participation. However, recent research suggests that the relationship between environmental awareness and gambling participation is more nuanced. Syvertsen et al. (2022) demonstrated that exposure to gambling-related cues increases general awareness among both gamblers and non-gamblers alike. This finding is further supported by Russell et al. (2018), who found that awareness of gambling opportunities extends beyond active participants through social network effects. Additionally, Tessier et al. (2021) highlighted how the normalisation of gambling in society influences awareness across different population segments, regardless of their gambling participation. These findings suggest that while self-reporting bias should be considered when interpreting our results, awareness of gambling venues is shaped by broader social and environmental factors that extend beyond individual gambling behaviour.

While the strategic placement of gambling venues in areas with higher revenue potential is well documented, our findings suggest a more nuanced relationship between venue location and actual encounters. Although operators typically locate their venues in areas with specific demographic characteristics associated with greater gambling propensities, this spatial strategy does not automatically translate into encounters during daily routines. Drawing from time-geography (Hägerstrand, 1970) and behavioural geography theory (Golledge & Stimson, 1997), we argue that the relationship between residential location and actual spatial behaviour is more complex. Individual activity spaces often extend beyond the immediate residential environment, and daily movement patterns are constrained by both spatial and temporal factors. Therefore, while the strategic placement of venues may influence overall gambling accessibility, the actual experience of encountering these venues depends on individual spatial behaviour patterns and routines rather than mere residential proximity.

## Conclusions

Consistent with extant literature, our empirical investigation corroborated a positive correlation between the spatial distribution of gambling facilities within municipalities and the frequency of their utilisation by local inhabitants. However, our findings indicate that a more robust predictor than the mere presence of gambling facilities in urban environments is the actual physical encounters with these facilities during residents'daily spatial movements. This observation elucidates a significant distinction between the availability of gambling activities within urban confines and their accessibility, which, in the context of our study, was operationalised as the frequency of physical encounters with land-based gambling facilities. The mobility patterns of residents, particularly in terms of commuting for employment and education, can substantially extend the accessibility of gambling facilities beyond the geographical boundaries of their place of residence. This phenomenon is particularly salient for facilities offering high-risk gambling forms, such as casino games, where the association between physical encounters and gambling frequency demonstrated the strongest correlation. These high-risk gambling facilities are frequently situated in larger urban centres, which also serve as regional hubs for daily migration flows.

This spatial configuration presents significant challenges for local governmental bodies in terms of implementing effective regulatory measures concerning the location of gambling facilities in high-traffic public areas, such as transportation hubs, central squares, and in proximity to educational institutions and recreational spaces. Furthermore, these findings underscore the necessity for more nuanced research endeavours to explore this complex interplay between spatial distribution, accessibility, and gambling behaviour in greater depth.

## Data Availability

The data that support the findings of this study are available from the corresponding author upon reasonable request.

## Footnotes

1 Evona Electronic, spol. s.r.o. (https://bet-x.cz/cs/terminal-locations), Fortuna Game a.s. (https://www.ifortuna.cz/mapapobocek?showAll=1), Chance a.s. (https://www.chance.cz/pobocky), Tipsport.net a.s. (https://www.tipsport.cz/pobocky).

2 Evona Electronic, spol. s.r.o. (https://bet-x.cz/cs/terminal-locations), Euro Queen a.s. (https://vyhernilosy.cz/), Fortuna Game a.s. (https://www.ifortuna.cz/mapapobocek?showAll=1), Loterie Korunka s.r.o. (https://www.korunka.eu/kde-hrat), SAZKA a.s. (https://www.sazka.cz/mapa), Tipsport a.s. (https://losy.tipsport.cz/prodejni-mista/).

3 In this sparsely populated municipality (according to the 2021 census, only 599 adult residents lived here) located in the hinterland of the regional centre, there was a casino at the beginning of 2023; however, no other gambling facility was present in the municipality.

4 Legal participation in gambling is allowed in the Czech Republic from the age of 18. For this reason, the survey focused exclusively on the adult population.

5 The specific land-based gambling activities for which respondents indicated their level of experience were as follows: numerical lotteries, scratch cards, sports betting, EGMs, live casino games, poker, and card tournaments. This list is derived from current legislation. For simplification, three categories were then utilised (lotteries, sports betting, casino games). For both lotteries (numerical lotteries and scratch cards) and casino games (EGMs, live casino games, poker, and card tournaments), we considered the most recent experience with any of the given gambling activities.

6 **Y –** gambling facility IS available in the respondent’s municipality; **NO –** gambling facility is NOT available in the respondent’s municipality; **E** – respondent DOES encounter gambling facilities; **NE** – respondent DOESN’T encounter gambling facilities.

7 More than 80% of these 161 respondents had experience with only one game and it was not in the last month. Therefore, it could probably be assumed that these are non-problem gamblers. Nevertheless, they were excluded from the further analysis as the authors cannot be sure of this assumption.
